# Structured shift huddle training and its influence on nurses’ knowledge, safety awareness, and clinical performance: a quasi-experimental investigation

**DOI:** 10.1186/s12912-026-05307-2

**Published:** 2026-09-11

**Authors:** Sara Sayed Abdel Wahab, Samah Faisal Fakhry, Nema Fathy Saad

**Affiliations:** 1 Faculty of Nursing, Ain Shams University, Cairo, Egypt; 2 Faculty of Nursing, May University, Cairo, Egypt

**Keywords:** Clinical training, Nursing practice, Patient safety goals, Safety awareness, Shift huddle, Situational awareness

## Abstract

**Background:**

Shift huddles are brief, structured team briefings used to create shared situational awareness, identify emerging safety concerns, and coordinate priorities. Evidence is generally supportive, but much of the published huddle literature uses uncontrolled pre-post designs, making causal attribution difficult. This study assessed changes in nurses’ knowledge, safety awareness, and clinical performance following a structured shift huddle and patient-safety training program.

**Methods:**

A one-group pretest-posttest quasi-experimental design was used in critical and non-critical inpatient units of a cardiovascular surgery teaching hospital affiliated with Ain Shams University Hospitals, Egypt. Eighty-two nurses (52 staff nurses and 30 supervisors/head nurses) completed baseline, immediate post-training, and follow-up assessments. Outcomes were measured using researcher-developed and adapted questionnaires and observational checklists covering shift huddles and International Patient Safety Goals (IPSGs).

**Results:**

Overall satisfactory knowledge increased from 32.9% at baseline to 93.9% immediately after training and 82.9% at follow-up. Overall high awareness increased from 29.3% to 100.0% and then 79.3%, respectively. Overall adequate patient-safety practice increased from 50.0% to 100.0% and then 75.6%. The largest changes were observed immediately after training, with partial retention at follow-up. Because the same nurses were measured repeatedly, the phase-comparison p-values from the source Pearson chi-square analysis are treated as exploratory and the descriptive changes are emphasized.

**Conclusions:**

Participation in the structured training program was associated with large short-term improvements in measured knowledge, awareness, and observed patient-safety practice, with partial attenuation at follow-up. Because the study had no concurrent control group and used direct observation, the magnitude of change cannot be attributed solely to the intervention. Controlled, multicentre evaluations with blinded or independently assessed outcomes are warranted.

**Trial registration:**

Not applicable.

**Supplementary Information:**

The online version contains supplementary material available at https://doi.org/10.1186/s12912-026-05307-2.

## Background

Safety huddles are brief, structured, recurring team conversations that focus attention on current or anticipated threats to safe care. Their defining functions include creating shared situational awareness, enabling team members to voice concerns, coordinating priorities, and supporting collective responsibility for risk management [1–4]. A huddle is conceptually distinct from a formal patient handover: handover transfers responsibility and detailed patient-specific information between outgoing and incoming clinicians, whereas a safety huddle is a short, team-level risk scan that may occur at the start of a shift or at other planned points and should complement rather than replace established handover processes [1–4].

Hospital huddles have been associated with improvements in selected teamwork, communication, safety-culture, and care-process outcomes, but the evidence base remains heterogeneous. A systematic review found that uncontrolled pre-post comparisons predominated and that rigorous controlled evidence was limited [2]. More recent studies have reported favorable results in intensive care and surgical settings [5–7], while other evaluations have shown only small or inconsistent changes in measured safety culture [8]. These mixed findings make intervention fidelity, measurement quality, and contextual reporting especially important.

Nurses are central to patient-safety surveillance because they continuously coordinate care, identify deterioration and process failures, administer medications, prevent infection and falls, and communicate across professional and shift boundaries. Knowledge, attitudes, team communication, standardization, education, and feedback have all been linked to nurses’ adherence to patient-safety principles [9–11]. Structured education that combines conceptual content with rehearsal of safety behaviors may therefore strengthen both cognitive readiness and day-to-day practice.

In the study hospital, baseline assessment suggested gaps in nurses’ knowledge of the huddle concept and in several patient-safety domains. The present study therefore evaluated a structured training program combining huddle education with practical application of the six IPSGs. Given the non-randomized, one-group design, the study was framed as an evaluation of within-participant change over time rather than a definitive test of causal effectiveness.

### Aim and hypothesis

The study aimed to assess changes in nurses’ knowledge, awareness, and clinical performance regarding shift huddles and international patient-safety goals before, immediately after, and at follow-up after a structured training program.

The study hypothesis was that post-training knowledge, awareness, and clinical-performance scores would be higher than baseline values.

## Methods

### Study design

A one-group pretest-posttest quasi-experimental design was used with three measurement phases: baseline, immediate post-training assessment, and follow-up. The absence of a concurrent comparison group was recognized a priori as a limitation on causal inference.

### Setting

The study was conducted across critical and non-critical inpatient units at the Cardiovascular Surgery Hospital affiliated with Ain Shams University Hospitals, Cairo, Egypt. Ten critical-care units comprising 65 beds were included: the Emergency Intensive Care Unit (ICU), Adult Cardiothoracic Surgery ICUs 1, 2, and 3, Pediatric ICU, Chest ICU, three Cardiac Care Units (CCU 1, CCU 2, and CCU 3), and the Post-Catheterization ICU. Three non-critical inpatient wards with a combined capacity of 86 beds were also included.

### Participants, sampling, and sample-size planning

The accessible population comprised 130 nurses employed in the participating units during the data-collection period. The study used a prespecified powered sample rather than attempting a census. The OpenEpi calculation compared planning proportions of 50% satisfactory knowledge at baseline and 75% after training, with alpha = 0.05 and 80% power, yielding a minimum sample of 66. A 20% allowance for attrition increased the target to 82, and all 82 enrolled nurses completed the study. The achieved sample included 52 staff nurses (63.4%) and 30 supervisors/head nurses (36.6%). Supervisors/head nurses were selected purposively, whereas staff nurses were selected using simple random sampling.

Eligible participants were nurses employed in one of the participating inpatient units during the data-collection period who agreed to participate and could complete the study assessments. The retained study documentation did not specify additional exclusion criteria; accordingly, no additional retrospective exclusions are claimed. Supervisors/head nurses were included purposively because of their unit-level coordination responsibilities, while staff nurses were selected by simple random sampling from the eligible staff-nurse roster. The specific random-number device used for selection was not retained in the study documentation.

For sample-size planning, a 50% baseline satisfactory-knowledge proportion was used as a conservative planning value and 75% was set as the anticipated post-training proportion, representing a 25-percentage-point improvement considered educationally meaningful for planning purposes. These values were prespecified for the OpenEpi calculation and were not derived from the nine-participant feasibility pilot, which was conducted later to assess instrument clarity and administration time.

### Data-collection instruments

Four instruments were used. Tool 1 combined a researcher-developed shift-huddle knowledge component with a previously published patient-safety knowledge component; Tool 2 and Tool 4 were developed for the present study; and Tool 3 was adapted from a previously published patient-safety performance checklist [12]. The provenance of each instrument is detailed below. The available English-language instrument documentation is provided in Supplementary File 1.

#### Tool 1: Shift huddle and patient safety goals knowledge questionnaire

This questionnaire contained three sections. Part 1 collected age, sex, educational qualification, department, years of experience, job position, and prior patient-safety training. Part 2 was developed specifically for the present study and contained 21 huddle-knowledge items (13 multiple-choice and 8 true/false) addressing the definition, timing, purposes, types, content, team roles, and procedural conduct of huddles. Item generation was informed by huddle literature and implementation guidance [1, 3, 4]. Part 3 contained 37 patient-safety knowledge items (21 multiple-choice and 16 true/false) adapted from the previously published instrument of Abd El Hamid et al. [12], covering the six IPSGs and safety-culture concepts. Correct answers were scored 1 and incorrect answers 0. A total percentage score of at least 60% was classified as satisfactory knowledge.

#### Tool 2: Patient safety goals awareness questionnaire

This 63-item questionnaire was developed specifically for the present study and was informed by published work on awareness and compliance with IPSGs [13, 14]. Items were distributed across Goal 1 (8 items), Goal 2 (11), Goal 3 (5), Goal 4 (15), Goal 5 (11), and Goal 6 (13). Responses used a five-point ordered scale scored in the direction of awareness (strongly aware = 5, aware = 4, neutral/moderately aware = 3, unaware = 2, strongly unaware = 1). Domain scores were converted to percentages so that higher values represented greater awareness; at least 60% was classified as high awareness.

#### Tool 3: Patient safety performance observational checklist

This 83-item checklist was adapted from the previously published patient-safety performance instrument of Abd El Hamid et al. [12] and assessed observed performance of IPSG-related nursing procedures. Each item was scored as done = 1 or not done = 0. A percentage score of at least 85% was classified as adequate performance.

#### Tool 4: Shift huddle performance observational checklist

This 41-item checklist was developed specifically for the present study from the huddle literature [1, 3, 4] and assessed execution of procedural steps in a structured shift huddle. Items were scored dichotomously (done = 1; not done = 0), and the same 85% adequacy threshold used for Tool 3 was applied for consistency.

The 60% knowledge/awareness and 85% performance thresholds were operational classification rules rather than diagnostic cut points. They were selected to align the scoring framework used in prior patient-safety educational work in comparable nursing populations, including the instrument from which the patient-safety components were adapted [12, 15].

Supplementary File 1 provides the available English-language documentation for all four tools, including provenance, domains, item counts, response formats, scoring rules, and citations for adapted or literature-informed components. The original item-by-item forms for the researcher-developed components were not retained in the documentation available to the authors; therefore, unverified wording has not been reconstructed or presented as if it were the original administered instrument.

### Instrument development, content validity, and reliability

For the researcher-developed components, candidate content domains were identified from the literature cited above and organized around the intended constructs (huddle knowledge, IPSG awareness, and huddle performance). Face and content validity were reviewed qualitatively by a five-member expert panel comprising three professors of nursing administration from the Faculty of Nursing, Ain Shams University, and two healthcare-quality experts from the Faculty of Medicine, Ain Shams University. The panel reviewed relevance, clarity, comprehensiveness, and wording; recommendations were incorporated before pilot testing. The retained study documentation does not report a numerical content-validity index or a prespecified number of iterative revision rounds, so no CVI value or unrecorded revision count is claimed.

Internal-consistency estimates in the study sample were high: knowledge questionnaire Cronbach’s alpha = 0.93, awareness questionnaire alpha = 1.00 (original value 0.997, reported to two decimals as requested), and performance checklists alpha = 0.95. The extremely high alpha for the awareness questionnaire should be interpreted cautiously because values near 1.00 can indicate substantial item similarity or redundancy; internal consistency alone does not establish construct validity or observer agreement.

### Pilot study

A pilot study was conducted in November 2022 with nine nurses (approximately 10% of the target sample) to assess clarity, completeness, and administration time. Questionnaire completion required approximately 25–30 min and each observation required approximately 40–45 min. No instrument or procedural modifications were required after the pilot. The nine pilot participants were therefore retained in the main sample because they had completed the same instruments under the same procedures as subsequent participants, avoiding the mixing of different instrument versions.

### Training program development and delivery

Baseline results were reviewed in January 2023 to identify knowledge and performance gaps and to guide development of the educational content, instructional booklet, and multimedia resources. Training was delivered during February-March 2023 in six 2-hour sessions (12 h total: 6 theoretical and 6 practical hours), with sessions scheduled on at least three mornings per week. Teaching strategies included mini-lectures, small-group discussion, brainstorming, case-based scenarios, demonstration and re-demonstration, role-play, instructional videos, and WhatsApp-supported learning. The six sessions covered: (1) the concept, definition, and benefits of shift huddles; (2) huddle structure, agenda, and documentation; (3) patient-safety concepts and the six IPSGs; (4) practical shift-huddle performance; (5) practical application of IPSGs 1–3; and (6) practical application of IPSGs 4–6. The research team used the same session objectives, core teaching sequence, and educational materials throughout delivery to standardize content across participants.

The training emphasized that a shift safety huddle is a brief team-level risk and coordination discussion and is not a substitute for formal patient-specific handover. Handover and huddle serve complementary purposes: handover transfers detailed responsibility for individual patients, whereas the huddle focuses the team on cross-cutting priorities, anticipated risks, resource issues, and escalation needs.

Training was organized around clinical duty schedules so nurses could complete the six-session curriculum without disrupting service delivery. All participants included in the post-training evaluation completed the planned educational content. Participation was reinforced through discussion, case scenarios, role-play, re-demonstration, videos, and WhatsApp-supported learning rather than through a separate quantitative engagement scale. The study records available for reporting did not specify a fixed participant number for each teaching group, session-by-session trainer credentials, a formal make-up-session log, a numerical fidelity score, or whether the booklet was distributed only in print, only electronically, or in both formats; these are acknowledged as limitations in intervention reporting.

The study intervention did not replace or formally modify patient-specific nursing handover. Huddle training focused on a complementary team-level safety and coordination process; detailed transfer of responsibility and patient-specific clinical information remained outside the intervention protocol.

### Data-collection procedure and timing

At baseline (December 2022), self-administered questionnaires were distributed during morning shifts between 09:00 and 13:00. Researchers remained available to clarify procedural questions. Each nurse was observed on three non-consecutive days separated by at least two days; observations lasted approximately 40–45 min and the mean of the three observations was used in the original analysis.

For the immediate post-training evaluation, the knowledge/awareness questionnaires were administered immediately after completion of the final training session. In contrast, the post-training observational assessment was not a single immediate observation: each nurse was observed three times during the subsequent one-month post-training observation window, again with at least two days between observations. This distinction clarifies the wording “immediate post-intervention.”

A follow-up assessment was conducted in August 2023 using the same questionnaires and observational approach.

Each nurse underwent three direct observations during each observational assessment period on non-consecutive days. Observations formed part of the consented study procedures, so participants were aware that clinical performance would be observed, although the exact observation schedule was not disclosed in advance. Formal observer blinding to study phase was not feasible in this sequential one-group design and cannot be assumed. The retained data-collection documentation does not provide a separate inter-rater agreement statistic; therefore, Cronbach’s alpha is reported only as an internal-consistency estimate and not as evidence of observer agreement. This limitation is addressed in the Discussion.

The final assessment is reported throughout as the August 2023 follow-up rather than assigning an exact month interval. This terminology reflects the documented chronology: training occurred during February-March 2023, post-training questionnaires were administered after the final session, post-training observations continued during the subsequent assessment window, and the final follow-up assessment was conducted in August 2023.

### Statistical analysis

Data were analyzed using IBM SPSS Statistics version 27. Categorical variables were summarized as frequencies and percentages and continuous variables as means with standard deviations or medians with ranges, as appropriate. The source analysis reported Pearson chi-square tests (with Fisher’s exact test when expected counts were small) for categorical phase comparisons, Spearman rank correlations for associations among scores, and multiple linear regression models for knowledge and awareness. Because the same nurses contributed measurements at more than one phase, the Pearson chi-square phase comparisons do not account for within-participant dependence; these p-values are therefore retained only as exploratory source analyses, while interpretation emphasizes descriptive within-cohort changes. Statistical significance in the original analysis was set at *p* < 0.05.

## Results

### Participant characteristics

Eighty-two nurses completed all study phases. Age ranged from 22 to 54 years (mean 38.8 ± 6.3 years); 53.7% were younger than 40 years and 54.9% were female. Fifty-two participants (63.4%) were staff nurses and 30 (36.6%) were supervisors/head nurses. Half worked in critical-care units, 23.2% in cardiac surgery, and 26.8% in general wards. The median nursing experience was 18 years (range 1–40), and 79.3% reported prior patient-safety training (Table 1).

**Table 1:** Demographic characteristics of the study sample (*n* = 82)

Characteristic	*n*	%
**Age (years)**		
< 40	44	53.7
≥ 40	38	46.3
Mean ± SD	38.8 ± 6.3	
Range	22–54	
**Sex**		
Female	45	54.9
Male	37	45.1
**Nursing qualification**		
Diploma (combined)	57	69.5
Bachelor’s degree	25	30.5
**Job position**		
Staff nurse	52	63.4
Supervisor/head nurse	30	36.6
**Department**		
Critical care units	41	50.0
Cardiac surgery	19	23.2
General wards	22	26.8
**Years of nursing experience**		
< 20	44	53.7
≥ 20	38	46.3
Median (range)	18 (1–40)	
**Prior patient safety training**		
Yes	65	79.3
No	17	20.7

### Knowledge of shift huddles and patient-safety goals

Overall satisfactory knowledge increased from 32.9% at baseline to 93.9% immediately post-training and 82.9% at follow-up. Total satisfactory huddle knowledge increased from 2.4% to 93.9% and 75.6%, and total patient-safety knowledge increased from 53.7% to 93.9% and 86.6%, respectively. The source Pearson chi-square analysis indicated large pre-to-post differences for most domains, but these p-values are interpreted exploratorily because the observations were repeated within the same nurses. At follow-up, not every individual domain showed the same pattern; for example, Goal 4 knowledge was 75.6% at baseline and 80.5% at follow-up (source *p* = 0.45) (Table 2).

**Table 2:** Knowledge of shift huddle and patient-safety goals across intervention phases (*n* = 82)

Domain	Pre *n*	Pre %	Post *n*	Post %	FU *n*	FU %	χ²	*p* (Pre–Post)	χ²	*p* (Pre–FU)
Huddle – Definition	8	9.8	77	93.9	72	87.8	116.28	< 0.001	99.96	< 0.001
Huddle – Timing	3	3.7	77	93.9	62	75.6	133.64	< 0.001	88.72	< 0.001
Huddle – Purpose	4	4.9	76	92.7	63	76.8	126.51	< 0.001	87.84	< 0.001
Huddle – Types	4	4.9	77	93.9	62	75.6	130.00	< 0.001	85.30	< 0.001
Huddle – Content	11	13.4	77	93.9	63	76.8	106.82	< 0.001	66.59	< 0.001
Huddle – Team roles	11	13.4	77	93.9	73	89.0	106.82	< 0.001	93.81	< 0.001
Huddle – Procedure	45	54.9	77	93.9	62	75.6	32.77	< 0.001	7.78	0.005
Total huddle (satisfactory)	2	2.4	77	93.9	62	75.6	137.38	< 0.001	92.25	< 0.001
PS – Definition	46	56.1	77	93.9	72	87.8	31.25	< 0.001	20.42	< 0.001
PS – General goals	51	62.2	77	93.9	73	89.0	24.06	< 0.001	16.00	< 0.001
PS – Goal 1	56	68.3	80	97.6	76	92.7	24.81	< 0.001	15.53	< 0.001
PS – Goal 2	44	53.7	82	100.0	68	82.9	49.46	< 0.001	16.22	< 0.001
PS – Goal 3	47	57.3	77	93.9	62	75.6	29.76	< 0.001	6.16	0.01
PS – Goal 4	62	75.6	82	100.0	66	80.5	22.78	< 0.001	0.57	0.45
PS – Goal 5	47	57.3	81	98.8	70	85.4	41.14	< 0.001	15.78	< 0.001
PS – Goal 6	46	56.1	82	100.0	72	87.8	46.13	< 0.001	20.42	< 0.001
PS – Safety culture	39	47.6	77	93.9	72	87.8	42.53	< 0.001	30.36	< 0.001
Total PS (satisfactory)	44	53.7	77	93.9	71	86.6	34.33	< 0.001	21.22	< 0.001
Overall total (satisfactory)	27	32.9	77	93.9	68	82.9	65.71	< 0.001	42.05	< 0.001

PS = patient safety; FU = follow-up; satisfactory ≥ 60%. Pearson chi-square p-values are reproduced from the source analysis and should be interpreted as exploratory because repeated measurements from the same participants are not independent

### Awareness of international patient-safety goals

At baseline, the proportion classified as highly aware was below 50% for every IPSG, ranging from 29.3% for Goal 6 to 46.3% for Goal 3. Immediately after training, 100% of nurses met the high-awareness threshold for each goal. At follow-up, the corresponding proportions ranged from 78.0% to 89.0%; overall high awareness was 79.3% compared with 29.3% at baseline (Table 3). The complete immediate post-training classification warrants cautious interpretation because measurement sensitivity, repeated testing, and heightened attention may have contributed to the observed ceiling.

**Table 3:** Nurses’ awareness of international patient-safety goals across intervention phases (*n* = 82)

IPSG	Pre *n*	Pre %	Post *n*	Post %	FU *n*	FU %	χ²	*p* (Pre–Post)	χ²	*p* (Pre–FU)
Goal 1 (Patient ID)	36	43.9	82	100.0	73	89.0	63.93	< 0.001	37.45	< 0.001
Goal 2 (Communication)	29	35.4	82	100.0	66	80.5	78.31	< 0.001	34.25	< 0.001
Goal 3 (Medications)	38	46.3	82	100.0	72	87.8	60.13	< 0.001	31.92	< 0.001
Goal 4 (Surgical safety)	26	31.7	82	100.0	65	79.3	85.04	< 0.001	37.55	< 0.001
Goal 5 (Infection control)	30	36.6	82	100.0	64	78.0	76.14	< 0.001	28.81	< 0.001
Goal 6 (Fall prevention)	24	29.3	82	100.0	64	78.0	89.74	< 0.001	39.23	< 0.001
Total (high awareness)	24	29.3	82	100.0	65	79.3	89.74	< 0.001	41.30	< 0.001

IPSG = International Patient Safety Goal; FU = follow-up; high awareness ≥ 60%. Pearson chi-square p-values are reproduced from the source analysis and should be interpreted as exploratory because repeated measurements from the same participants are not independent

### Clinical performance of patient-safety goals

Overall adequate observed practice increased from 50.0% at baseline to 100.0% in the post-training observation period and 75.6% at follow-up. The source analysis reported large pre-to-post and pre-to-follow-up differences for the outcomes shown in Table 4, but the Pearson chi-square p-values are interpreted exploratorily because observations were repeated within the same participants. The source clinical-performance table provided values for Goals 1, 2, 3, 5, and 6 and did not report a separate Goal 4 (surgical safety) performance row; therefore, the present report restricts domain-specific performance claims to the five reported goals and does not impute a Goal 4 value.

**Table 4:** Nurses’ clinical performance of patient-safety goals across intervention phases (*n* = 82)

Goal / Outcome	Pre *n*	Pre %	Post *n*	Post %	FU *n*	FU %	χ²	*p* (Pre–Post)	χ²	*p* (Pre–FU)
Goal 1 (Patient ID) – adequate	41	50.0	81	98.8	62	75.6	51.21	< 0.001	11.51	0.001
Goal 2 (Communication) – adequate	42	51.2	82	100.0	62	75.6	52.90	< 0.001	10.51	0.001
Goal 3 (Medications) – adequate	41	50.0	82	100.0	63	76.8	54.67	< 0.001	12.72	< 0.001
Goal 5 (Infection control) – adequate	42	51.2	82	100.0	63	76.8	52.90	< 0.001	11.68	0.001
Goal 6 (Fall prevention) – adequate	44	53.7	82	100.0	64	78.0	49.46	< 0.001	10.85	0.001
Overall adequate practice	41	50.0	82	100.0	62	75.6	54.67	< 0.001	11.51	0.001

Adequate performance ≥ 85%; FU = follow-up. Goal 4 was not reported as a separate clinical-performance row in the source results; no value was imputed. Pearson chi-square p-values are reproduced from the source analysis and should be interpreted as exploratory because repeated measurements from the same participants are not independent

### Correlations among knowledge, awareness, and practice

The reported Spearman coefficients were rho = 0.360 between knowledge and practice, rho = 0.256 between knowledge and the awareness-score difference, and rho = 0.199 between the awareness-score difference and practice (Table 5). These coefficients indicate small-to-moderate positive monotonic relationships. Because exact correlation p-values could not be independently verified from the available summary outputs, Table 5 reports the coefficients without unverified significance asterisks.

**Table 5:** Correlation matrix of knowledge, awareness, and patient-safety practice scores (*n* = 82)

Variable	Knowledge score	Awareness score difference	Practice score
Knowledge score	1.000		
Awareness score difference	0.256	1.000	
Patient safety practice score	0.360	0.199	1.000

Spearman’s rho coefficients are shown without significance asterisks because exact p-values depend on the participant-level ranks and tie structure, which were not available in the reported summary outputs

### Multivariable models

In the originally reported knowledge-score model (R² = 0.91), the training-phase indicator, age, and prior patient-safety training were included as predictors. In the awareness-score model (R² = 0.39), the training-phase indicator, age, sex, qualification level, and current experience were included. Tables 6 and 7 label the unstandardized multivariable coefficient as adjusted B and present its 95% confidence interval, as requested by the editor. Because the reported model specification does not document an explicit repeated-measures correlation structure for phase, these coefficients are presented as exploratory adjusted associations and are not interpreted as causal intervention effects.

**Table 6:** Multiple linear regression model for knowledge score (R² = 0.91)

Predictor	Adjusted B	SE	β	t	*p*	95% CI for adjusted B
Constant	17.28	8.39	—	2.060	0.041	0.71 to 33.85
Training intervention	43.96	2.40	0.82	18.339	< 0.001	39.23 to 48.69
Age (years)	−0.45	0.19	−0.11	−2.324	0.021	−0.84 to − 0.07
Prior safety training courses	7.08	3.01	0.11	2.354	0.020	1.14 to 13.03

**Table 7:** Multiple linear regression model for awareness score (R² = 0.39)

Predictor	Adjusted B	SE	β	t	*p*	95% CI for adjusted B
Constant	118.03	23.46	—	5.031	< 0.001	71.69 to 164.37
Training intervention	21.07	2.31	0.57	9.142	< 0.001	16.52 to 25.63
Age (years)	0.37	0.20	0.12	1.786	0.076	−0.04 to 0.77
Female sex	−4.38	2.33	−0.12	−1.880	0.062	−8.99 to 0.22
Qualification level	5.21	2.60	0.13	2.004	0.047	0.07 to 10.34
Current experience (years)	−7.24	2.55	−0.19	−2.833	0.005	−12.28 to − 2.19

## Discussion

The study observed large increases in measured huddle/patient-safety knowledge, awareness, and observed practice after a multimodal training program, followed by partial attenuation at follow-up. These findings are consistent with an educational effect but should not be interpreted as proof that the program alone caused the changes. In a one-group pretest-posttest design, repeated testing, maturation, contemporaneous hospital initiatives, peer diffusion, increased researcher attention, and observation-related behavior change can produce or amplify apparent pre-post differences.

The particularly low baseline huddle knowledge is plausible in a setting where structured huddles were not yet embedded as a routine team process. The combination of short didactic content, case discussion, demonstration, re-demonstration, role-play, videos, and mobile reinforcement provided repeated opportunities for encoding and retrieval of huddle concepts. Similar nursing-education studies have reported substantial post-training gains in patient-safety knowledge and performance [12, 15–17]. Nevertheless, educational improvement in questionnaire performance is not synonymous with durable behavior change; the decline at follow-up in the present study reinforces the need for reinforcement and workplace integration.

Huddles may influence safety behavior through mechanisms that extend beyond knowledge acquisition. A well-run huddle creates a shared mental model of the shift, increases situational awareness, legitimizes speaking up about risk, makes dependencies and assistance needs visible, and provides a recurring coordination routine. These mechanisms are reflected in concept analyses and implementation studies [1, 3, 5–7]. In a randomized study, proactive huddles reduced missed nursing care relative to control wards [7], supporting the plausibility that structured team coordination can affect care processes. However, a systematic review of hospital safety huddles concluded that most earlier evidence was uncontrolled and that implementation and reporting varied substantially [2]. Wahl et al. also found mostly minor or no changes in safety-culture measures despite generally positive staff experiences [8]. The present results should therefore be interpreted as supportive but not definitive evidence.

The 100% immediate post-training awareness classifications and near-universal adequate practice deserve particular scrutiny. Ceiling values can occur after intensive, criterion-referenced training, but they can also reflect broad cutoffs, highly similar questionnaire items, repeated exposure to the same instruments, limited measurement discrimination, or observer expectancy. The awareness scale’s alpha of 1.00 after rounding further suggests that some items may be highly redundant. Future validation should examine item-total behavior, dimensionality, test-retest properties, and sensitivity to change rather than relying on Cronbach’s alpha alone.

Direct observation introduces an additional risk of reactivity. Awareness of being observed can temporarily change clinician behavior, a phenomenon commonly discussed as the Hawthorne effect [18, 19]. This is especially relevant to the immediate post-training performance result of 100%. Repeated observations on non-consecutive days improve sampling of behavior, but they do not remove observer bias or reactivity. Blinded assessment, independent observers, unobtrusive process indicators, and objective patient-safety outcomes would strengthen future evaluations.

The modest decline by follow-up is also informative. Huddle and safety behaviors are social routines that depend on leadership support, staffing, protected time, psychological safety, feedback, and repeated use. Education may initiate the behavior, but organizational reinforcement is needed to sustain it. The systematic review by Vaismoradi et al. similarly identified education, feedback, collaboration, standardization, and work-environment factors as determinants of nurses’ adherence to safety principles [10].

The sample included both staff nurses and supervisors/head nurses, whose baseline exposure to safety governance and leadership responsibilities may differ. Job position could therefore influence baseline knowledge or responsiveness to training. The reported summary data do not permit a reliable post hoc position-stratified outcome analysis; consequently, the mixed-role composition is treated as a limitation and future studies should prespecify subgroup analyses or stratified designs where appropriate.

### Practical and policy implications

The findings support testing structured huddle education as part of a broader implementation strategy rather than as a stand-alone course. Hospitals considering adoption should define a brief standard huddle agenda, clarify its relationship to formal handover, prepare huddle leaders, provide protected time, monitor fidelity, and use periodic feedback or refresher activities. Managers should avoid treating high post-training scores as sufficient evidence of sustained practice; routine audit should include process measures and, where feasible, patient-level safety outcomes. At policy level, huddle competencies can be incorporated into orientation and continuing professional development while preserving unit flexibility for local risk priorities.

### Strengths and limitations

Strengths include assessment across critical and non-critical cardiovascular units, measurement of cognitive and observed practice domains, repeated observations of each nurse within each observational phase, and inclusion of a follow-up assessment rather than a baseline/immediate-post design only. The multimodal intervention was also described in sufficient broad detail to identify its educational components.

Several limitations materially affect interpretation. First, the single-group quasi-experimental design cannot separate intervention-associated change from secular trends, repeated testing, regression to the mean, accreditation or institutional activities, or other co-interventions. Second, direct observation may have generated Hawthorne effects and observer expectancy, especially given the ceiling post-training performance. Third, formal observer blinding was not feasible and a separate inter-rater agreement statistic was not reported; Cronbach’s alpha does not establish observer agreement. Fourth, the awareness instrument showed an alpha of 1.00 after rounding, which may reflect item redundancy and limits confidence that the scale captures a broad, discriminating construct. Fifth, supervisors/head nurses and staff nurses were combined, and job-position differences were not modeled in the reported outcomes. Sixth, all participants came from one specialized teaching hospital, limiting external generalizability. Seventh, the source Pearson chi-square phase comparisons did not account for the repeated nature of the measurements and are therefore interpreted as exploratory rather than definitive inferential tests. Eighth, the reported regression specification did not document a repeated-measures correlation structure, so adjusted phase coefficients should be interpreted cautiously. Finally, the source clinical-performance table did not separately report Goal 4; performance conclusions are consequently restricted to the reported domains.

## Conclusions

Participation in the structured shift huddle and patient-safety training program was associated with marked improvements in measured knowledge, awareness, and observed safety practice immediately after training, with partial retention at follow-up. The findings are educationally promising but do not establish a causal effect because there was no concurrent control group and several outcomes reached ceiling values under direct observation. Future controlled, multicentre studies should use validated and discriminating measures, clearly documented intervention-fidelity procedures, appropriate paired or longitudinal analyses, and patient-level safety outcomes where feasible.

## Supplementary Information

Below is the link to the electronic supplementary material.

Supplementary Material 1

## Data Availability

The datasets used and/or analysed during the current study are available from the corresponding author on reasonable request, subject to applicable institutional and ethical requirements.

## Abbreviations

CCU: Cardiac Care Unit
FU: Follow-up
ICU: Intensive Care Unit
IPSG: International Patient Safety Goal
PS: Patient safety
SD: Standard deviation
SPSS: Statistical Package for the Social Sciences
