# Supplementary material for: Structured shift huddle training and its influence on nurses’ knowledge, safety awareness, and clinical performance: a quasi-experimental investigation
Source: BMC Nurs. 2026 Sep 11;25:852. doi: 10.1186/s12912-026-05307-2 (PMC13579718; doi:10.1186/s12912-026-05307-2)
Supplement: Supplementary file 1 [file 12912_2026_5307_MOESM1_ESM.docx]

**Supplementary File 1**

**English-language instrument documentation and scoring specification**

Structured Shift Huddle Training and Its Influence on Nurses’ Knowledge, Safety Awareness, and Clinical Performance: A Quasi-Experimental Investigation

**Important documentation note.** This file reports the English-language instrument information that is verifiable from the retained study documentation. It distinguishes components developed specifically for the present study from components adapted from published work. The original item-by-item forms for the researcher-developed components were not retained in the documentation available to the authors. To avoid research-record reconstruction, no unverified item wording is presented as a verbatim copy of the instruments administered in the study. If the original completed or blank English-language forms are recovered, they should replace or be appended to this specification before final publication.

# 1. Instrument provenance at a glance

| **Instrument** | **Provenance** | **Source / development basis** | **Length** |
| --- | --- | --- | --- |
| Tool 1: Shift Huddle and Patient Safety Goals Knowledge Questionnaire | Mixed provenance | Part 1: demographic data; Part 2: developed for this study; Part 3: adapted from Abd El Hamid et al. (2018) [S4]. | 58 scored knowledge items plus demographic section |
| Tool 2: Patient Safety Goals Awareness Questionnaire | Developed for this study | Developed for the present study; informed by IPSG awareness/compliance literature [S5,S6]. | 63 items |
| Tool 3: Patient Safety Performance Observational Checklist | Adapted from published work | Adapted from Abd El Hamid et al. (2018) [S4]. | 83 items |
| Tool 4: Shift Huddle Performance Observational Checklist | Developed for this study | Developed for the present study from huddle literature and implementation guidance [S1-S3]. | 41 items |

# 2. Tool 1: Shift Huddle and Patient Safety Goals Knowledge Questionnaire

**Part 1 - Participant characteristics.** Age, sex, educational qualification, department, years of experience, job position, and previous patient-safety training.

**Part 2 - Shift-huddle knowledge.** Developed specifically for the present study. The documented form contained 21 items: 13 multiple-choice questions and 8 true/false questions. Content domains were definition, timing, purposes, types, content, team roles, and procedural conduct of shift huddles. Item development was informed by huddle literature and implementation guidance [S1-S3]. Domain-specific item counts and the original item wording were not retained in the available study record.

**Part 3 - Patient-safety knowledge.** Adapted from the previously published instrument of Abd El Hamid et al. [S4]. The documented form contained 37 items: 21 multiple-choice questions and 16 true/false questions covering the six International Patient Safety Goals (IPSGs) and safety-culture concepts.

**Scoring.** Correct answer = 1; incorrect answer = 0. Scores were converted to percentages. A total percentage score >=60% was classified as satisfactory knowledge.

# 3. Tool 2: Patient Safety Goals Awareness Questionnaire

Developed specifically for the present study and informed by published work on awareness and compliance with IPSGs [S5,S6]. The questionnaire contained 63 items distributed as follows:

| **IPSG domain** | **Number of items** |
| --- | --- |
| Goal 1 - Patient identification | 8 |
| Goal 2 - Effective communication | 11 |
| Goal 3 - Medication safety | 5 |
| Goal 4 - Surgical safety | 15 |
| Goal 5 - Infection prevention/control | 11 |
| Goal 6 - Fall prevention | 13 |

**Response scale.** Strongly aware = 5; aware = 4; neutral/moderately aware = 3; unaware = 2; strongly unaware = 1.

**Scoring.** Domain and total scores were converted to percentages so that higher scores indicated greater awareness. A percentage score >=60% was classified as high awareness. The original item-by-item wording was not retained in the available study record.

# 4. Tool 3: Patient Safety Performance Observational Checklist

An 83-item observational checklist adapted from Abd El Hamid et al. [S4] to assess observed nursing performance of procedures related to the International Patient Safety Goals. The retained documentation confirms coverage of IPSG-related nursing procedures but does not preserve the complete item-by-item wording in a form that can be reproduced verbatim here.

**Scoring.** Done = 1; not done = 0. Scores were converted to percentages. A percentage score >=85% was classified as adequate performance.

# 5. Tool 4: Shift Huddle Performance Observational Checklist

Developed specifically for the present study from huddle literature and implementation guidance [S1-S3]. The checklist contained 41 items assessing procedural execution of a structured shift huddle. The available study record identifies the construct, length, and scoring method, but the original item-by-item wording was not retained.

**Scoring.** Done = 1; not done = 0. Scores were converted to percentages. A percentage score >=85% was classified as adequate performance.

# 6. Validity and reliability information reported for the study instruments

For researcher-developed components, face and content validity were reviewed qualitatively by a five-member expert panel: three professors of nursing administration from the Faculty of Nursing, Ain Shams University, and two healthcare-quality experts from the Faculty of Medicine, Ain Shams University. The panel reviewed relevance, clarity, comprehensiveness, and wording. No numerical content-validity index was retained in the study documentation.

Internal-consistency estimates reported in the study sample were: knowledge questionnaire Cronbach’s alpha = 0.93; awareness questionnaire alpha = 0.997 (reported as 1.00 when rounded to two decimals); and performance checklists alpha = 0.95. These values reflect internal consistency and should not be interpreted as inter-rater agreement for the observational checklists.

# 7. Operational classification thresholds

| **Outcome** | **Threshold** | **Interpretation** |
| --- | --- | --- |
| Knowledge | >=60% | Satisfactory |
| Awareness | >=60% | High awareness |
| Observed performance | >=85% | Adequate |

These were operational study classification rules rather than diagnostic cut points.

# References for instrument provenance and development

S1. Ghoul I, Abdullah A, Awwad F, Dardas LA. Safety huddle in healthcare settings: a concept analysis. BMC Health Serv Res. 2025;25:393. doi:10.1186/s12913-025-12526-x.

S2. Goldenhar LM, Brady PW, Sutcliffe KM, Muething SE. Huddling for high reliability and situation awareness. BMJ Qual Saf. 2013;22(11):899-906. doi:10.1136/bmjqs-2012-001467.

S3. Shaikh U. Improving patient safety and team communication through daily huddles. PSNet, Agency for Healthcare Research and Quality. 2020 Aug 19.

S4. Abd El Hamid LA, Shazly MM, Fakhry SF, Saad NF. Self-Learning Package Related to Patient Safety Goals and its effect on Nurse Interns’ Performance and Awareness of Patient Safety Culture. Egyptian Journal of Health Care. 2018;9(1):316-333. doi:10.21608/ejhc.2018.46499.

S5. Omer JA, Al-Rehaili OA, Al-Johani H, Alshahrani D. Residents’ Awareness about International Patient Safety Goals, Cross Sectional Study. Archives of Pediatrics. 2018;3:139. doi:10.29011/2575-825X.100039.

S6. Shahin MAHA, Alshammari RK, Alabed HH. Quality of Care and Patients’ Safety Awareness and Compliance among Critical Care Nurses at Qassim National Hospital: Adopting IPSGs. IOSR J Nurs Health Sci. 2020;9(3 Series VIII):1-11. doi:10.9790/1959-0903080111.
